# Collagen fragments quantified in serum as measures of desmoplasia associate with survival outcome in patients with advanced pancreatic cancer

**DOI:** 10.1038/s41598-019-56268-3

**Published:** 2019-12-24

**Authors:** Nicholas Willumsen, Suhail M. Ali, Kim Leitzel, Joseph J. Drabick, Nelson Yee, Hyma V. Polimera, Vinod Nagabhairu, Laura Krecko, Ayesha Ali, Ashok Maddukuri, Prashanth Moku, Aamnah Ali, Joyson Poulose, Harry Menon, Neha Pancholy, Luis Costa, Morten A. Karsdal, Allan Lipton

**Affiliations:** 1 Biomarkers & Research, Nordic Bioscience, Herlev, Denmark; 2 Division of Hematology/Oncology, Penn State Health Milton S Hershey Medical Center, Hershey, PA USA; 3 Lebanon VA Medical Center, Lebanon, PA USA; 4 Pinnacle Health System, University of Pittsburgh Medical Center, Harrisburg, PA USA; 5 Oncology division, Hospital de Santa Maria, Lisboa, Portugal; 6 Clinical Translational Oncology Research Unit, Institute of Molecular Medicine, Lisboa, Portugal

**Keywords:** Tumour biomarkers, Post-translational modifications

## Abstract

Pancreatic ductal adenocarcinoma (PDAC) patients have poor prognosis and poor response to treatment. This is largely due to PDAC being associated with a dense and active stroma and tumor fibrosis (desmoplasia). Desmoplasia is characterized by excessive degradation and formation of the extracellular matrix (ECM) generating collagen fragments that are released into circulation. We evaluated the association of specific collagen fragments measured in pre-treatment serum with outcome in patients with PDAC. Matrix metalloprotease (MMP)-degraded type I collagen (C1M), type III collagen (C3M), type IV collagen (C4M) and a pro-peptide of type III collagen (PRO-C3) were measured by ELISA in pre-treatment serum from a randomized phase 3 clinical trial of patients with stage III/IV PDAC treated with 5-fluorouracil based therapy (n = 176). The collagen fragments were evaluated for their correlation (r, Spearman) with serum CA19-9 and for their association with overall survival (OS) based on Cox-regression analyses. In this phase 3 PDAC trial, pre-treatment serum collagen fragment levels were above the reference range for 67%-98% of patients, with median values in PDAC approximately two-fold higher than reference levels. Collagen fragment levels did not correlate with CA19-9 (r = 0.049–0.141, *p* = *ns*). On a continuous basis, higher levels of all collagen fragments were associated with significantly shorter OS. When evaluating degradation (C3M) and formation (PRO-C3) of type III collagen further, higher PRO-C3 was associated with poor OS (>25^th^ percentile cut-point, HR = 2.01, 95%CI = 1.33–3.05) and higher C3M/PRO-C3 ratio was associated with improved OS (>25^th^ percentile cut-point, HR = 0.53, 95%CI = 0.34–0.80). When adjusting for CA19–9 and clinical covariates, PRO-C3 remained significant (HR = 1.65, 95%CI = 1.09–2.48). In conclusion, collagen remodeling quantified in pre-treatment serum as a surrogate measure of desmoplasia was significantly associated with OS in a phase 3 clinical PDAC trial, supporting the link between desmoplasia, tumorigenesis, and response to treatment. If validated, these biomarkers may have prognostic and/or predictive potential in future PDAC trials.

## Introduction

Pancreatic cancer (PC) is one of the most lethal types of cancer with an overall 5-year survival rate of 8% dropping to 3% in the metastatic setting^1^. At time of diagnosis, approximately 10% of PC patients have tumors that are potentially curable with resection, and 50% have metastatic disease. In 2017, in the United States alone, 55,440 new cases and 44,330 deaths were recorded. It has been projected that PC will become the second leading cause of cancer-related death in the United States in the next decade, hence PC is a clear exception from the general trend of improvement in survival rates for patients with other cancers^1,2^.

Not only is PC diagnosed late, but patients with advanced PC also respond poorly to chemo- and targeted-therapies^3,4^. Currently, besides imaging (computed tomography), carbohydrate antigen 19-9 (CA19-9), an antigen released by pancreatic cancer cells and measured in serum, is the only recommended biomarker for predicting and monitoring outcome in the PC setting^5^. Taken together, the grim PDAC outcome emphasizes the need not only for innovative drug development efforts, but also for the development of biomarkers that can be used for stratifying PC patients and predicting their outcome and likelihood of response to treatment.

Pancreatic ducal adenocarcinoma (PDAC) is the most common form of PC and accounts for more than 80% of cases. PDAC is the most stromal cancer type across all tumor indications, characterized by an extremely dense and desmoplastic (fibrotic) tumor microenvironment^6,7^. The desmoplastic reaction is highly associated with therapy resistance and tumorigenesis, thus accounting for the lack of satisfactory outcomes for PDAC patients^8,9^.

Desmoplasia is characterized by an altered remodeling of the collagenous extracellular matrix (ECM), which is the non-cellular component of tissue. While the ECM turnover in healthy tissue is maintained in a delicate homeostatic state between ECM/collagen formation and degradation, this homeostasis is lost in the tumor tissue^10–13^. Upregulation of the production and assembly of collagens, i.e. the formation of a fibrous connective tissue, is driven by proliferation and activation of myofibroblasts and myofibroblast-like stellate cells^14,15^. An associated collagen degradation is concomitantly observed, and is driven by a highly proteolytically-active tumor microenvironment, in which the matrix metalloproteases (MMPs) play a key role in degrading collagens^16,17^.

The interstitial matrix-associated type I collagen, type III collagen, and the basement membrane-associated type IV collagen have all been found upregulated in the primary tumors and metastatic lesions of PDAC, and are associated with poor outcome and pro-tumorigenic events^7,18–21^. Type IV collagen is a network forming collagen that supports the epithelia/endothelia whereas type I and type III collagen are fibrillar collagens part of the underlying stromal interstitial matrix. While tumor cell invasion and angiogenesis are associated with remodeling and degradation of type IV collagen, the synthesis, deposition, remodeling and degradation of the fibrillar collagens is more directly linked to fibroblast activity^21–25^.

A pilot study has evaluted the biomarker potential of specific MMP-derived fragments of type I (C1M), type III (C3M), and type IV (C4M) collagen as surrogate measures of desmoplasia and ECM remodeling, and found that all these fragments were elevated in serum from patients with PDAC compared to healthy controls^26^. Based on these findings, and the importance of desmoplasia in PDAC, we hypothesized that C1M, C3M, C4M, as well as PRO-C3, a biomarker derived from the pro-peptide of type III collagen, were predictive of outcome in the PDAC setting.

The aim of this study was to evalute how levels of specific collagen fragments (C1M, C3M, C4M, PRO-C3), quantified in pre-treatment serum, were associated with overall survival (OS) in a randomized phase 3 clinical trial of patients with advanced unresectable PDAC. The association between the collagen markers and CA19-9, the gold-standard PDAC serum biomarker, and outcome was also evaluated.

## Methods

### Patients and study design

The present study includes 176 patients with advanced unresectable PDAC that were enrolled in a randomized, double-blinded, placebo-controlled, multicenter phase III trial. One arm received octreotide (somatostatin, a somatotrophin-release inhibiting factor analogue) and continuous infusion of 5-fluorouracil (5-FU) and another arm received placebo and 5-FU^27^.

Briefly, patient inclusion criteria included (1) at least 18 years of age; (2) measurable or evaluable, stage III or IV, pathologically, histologically, or cytologically confirmed unresectable adenocarcinoma of the exocrine pancreas; and (3) no prior chemotherapy, radiation, or hormonal therapy^28^. The trial was powered to achieve 330 evaluable patients (165 per arm) with planned randomization of 412 patients (206 per arm) to detect an increase in 1-year survival rate from 10% in the 5-FU + placebo group to 20% in the 5-FU + octreotide group with a power of 0.85. The primary objective of this trial was overall survival (OS); secondary objectives were progression-free survival (PFS), clinical benefit, tolerability of octreotide, and objective response rate. Treatment after randomization was octreotide (160 mg intramuscular initiation, every 2 weeks for four injections and then every 4 weeks until disease progression) vs. placebo; 5-FU was given immediately following the first octreotide injection, by continuous intravenous infusion at a total daily dose of 225 mg/m2, for 8 weeks, followed by a 7-day rest period, and then, 5-FU was repeated until disease progression. The trial was stratified for gender, Karnofsky performance status (KPS, 70–100% vs. 50–60%), and stage of disease (III vs. IV).

The interim efficacy analysis of this clinical trial reported that the median OS time in the octreotide arm was 22.6 weeks (95%CI: 18.1–27.7) vs. 21.6 weeks in the placebo arm (95%CI: 17.9–28.3); this was not a significant difference (P = 0.649) in OS between the two arms (27). There was one complete (stage III, in octreotide arm) and two partial remissions (both in stage IV; one in octreotide arm and one in placebo arm). Since there was no efficacy difference in the two arms of this trial, it is optimal for biomarker discovery and validation. This same cohort had previously been used to report a significant association between higher inflammatory serum biomarkers (ferritin and C-reative protein, CRP) and shorter OS^28^.

### Serum biomarker measurements

Biomarker levels were measured in pre-treatment serum samples. C1M (MMP-degraded type I collagen, originally described by Leeming *et al*.^29^), C3M (MMP-degraded type 3 collagen, originally described by Veidal *et al*.^30^), C4M (MMP-degraded type IV collagen, originally described by Veidal *et al*.^31^) and PRO-C3 (N-terminal pro-peptide of type III collagen, originally described by Nielsen *et al*.^32^) were quantified by individual competitive enzyme-linked immune sorbent associated assays (ELISAs) according to the manufacturer’s instructions (Nordic Bioscience, Herlev, Denmark). The normal reference range (mean with 95%CI) for the collagen markers were pre-defined from a population of 617 healthy men and women ranging from 22 to 86 years of age^33^. The upper limit of the reference range was 28.4 ng/ml for C1M, 10.3 ng/ml for C3M, 19.3 ng/ml for C4M and 8.7 ng/ml for PRO-C3. Biomarker values below/above the detection limit were assigned the lower/upper limit of detection value for each respective assay.

### Statistical analysis

The serum collagen fragments (C1M, C3M, C4M and PRO-C3) were analyzed for an association with OS by use of univariate and multivariate Cox proportional hazards modeling. Biomarkers (variables) were evaluated both on a continuous and categorical scale, the latter by dichotomizing the variables by separation at the 25^th^ percentile cut-point. The dichotomized variables were also evaluated by Kaplan-Meier survival estimates (OS curves). Correlations between serum collagen fragments and CA19-9 were evaluated by calculating the Spearman rank correlation coefficients. A p-value < 0.05 was considered statistically significant.

### Ethics approval and consent to participate

Signed informed consent to participate in the present study was obtained from all patients before sample collection. This study was reviewed and approved by the institutional review boards at the Pennsylvania State University Hershey Medical Center and performed in accordance with the Declaration of Helsinki^28^.

## Results

### Patient demographics

The demographics and patient characteristics are shown in Supplementary Tables 1 and 2.

### Levels of collagen fragments in serum from patients with PDAC

**Figure 1:**
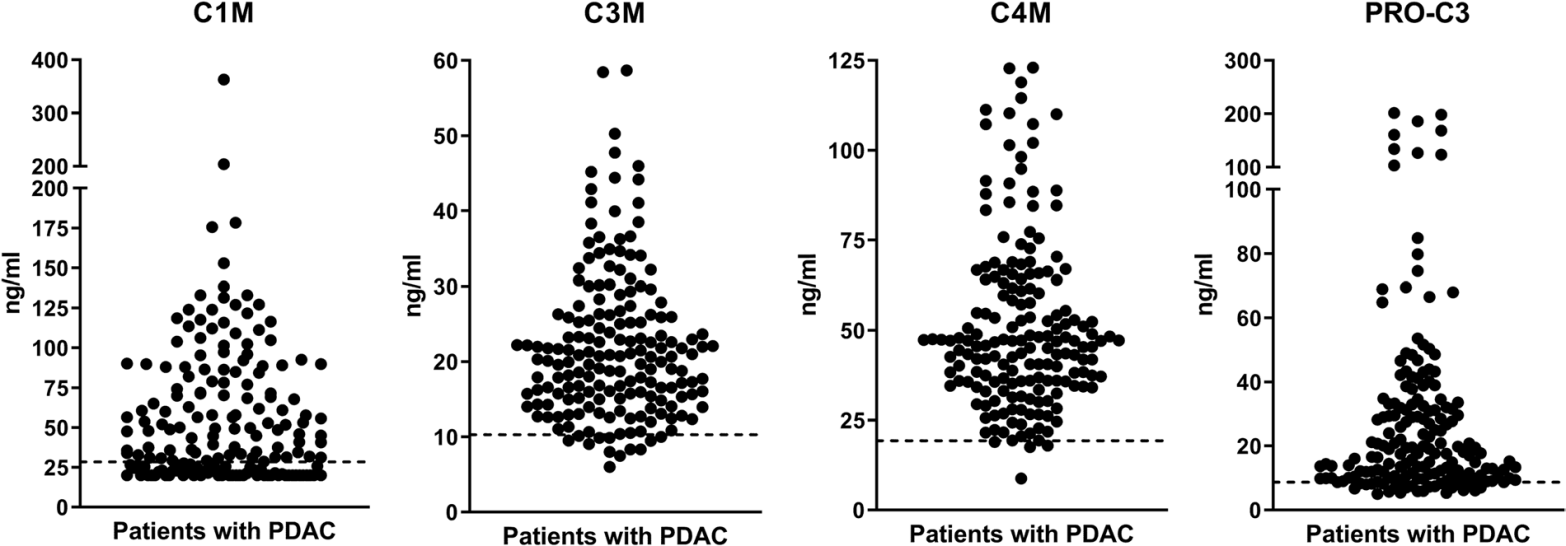
Levels of collagen fragments (C1M, C3M, C4M, PRO-C3) in pre-treatment serum from 176 patients with advanced pancreatic ductal adenocarcinoma (PDAC). C1M measures MMP-degraded type I collagen, C3M measures MMP-degraded type 3 collagen, C4M measures MMP-degraded type IV collagen, and PRO-C3 measures the N-terminal pro-peptide of type III collagen. The dotted line represents the upper limit of the normal reference range (defined as mean with 95%CI) from a population of 617 healthy men and women ranging from 22 to 86 years of age^33^.

An overview of the distribution of the collagen fragments C1M, C3M, C4M and PRO-C3 in pre-treatment serum samples from 176 patients with advanced PDAC is shown in Table 1 and Fig. 1. Overall, most of patients presented with biomarker levels above the reference range and with median values in PDAC approximately two-fold higher than the reference range.

**Table 1:** Overview of collagen fragment biomarker levels in pre-treatment serum from patients with pancreatic ductal adenocarcinoma (PDAC).

	C1M	C3M	C4M	PRO-C3
Minimum, ng/ml	20.0	6.0	8.8	5.0
25% Percentile, ng/ml	23.4	15.1	35.7	10.4
Median, ng/ml	44.7	20.5	46.9	17.7
75% Percentile, ng/ml	84.4	26.3	64.0	33.1
Maximum, ng/ml	362.9	58.7	123.0	201.8
% above reference range	67%	93%	98%	86%

### Correlations between collagen fragments and CA19-9

Table 2 shows the Spearman rank correlation coefficients (r) between the collagen markers (C1M, C3M, C4M and PRO-C3) and CA19-9. While all collagen fragments correlated with each other (r = 0.462–0.897, *p* < *0*.*0001*), no correlation was seen between any of the collagen markers and CA19-9 (r = 0.049–0.141, *p* = *0*.*065–0*.*521*). This indicates that the collagen fragments represent similar pathological events, and that these are distinct from the pathological events linked to CA19-9.

**Table 2:** Spearman rank correlation coefficients (r) between the collagen markers (C1M, C3M, C4M and PRO-C3) and CA19-9.

		C3M, ng/ml	C4M, ng/ml	PRO-C3, ng/ml	CA 19-9, U/ml
C1M, ng/ml	r *p-value*	0.679<*0*.*0001*	0.636<*0*.*0001*	0.485<*0*.*0001*	0.084*0*.*2751*
C3M, ng/ml	r *p-value*	— —	0.897<*0*.*0001*	0.462<*0*.*0001*	0.049*0*.*5213*
C4M, ng/ml	r *p-value*		— —	0.516<*0*.*0001*	0.115*0*.*1297*
PRO-C3, ng/ml	r *p-value*			— —	0.141*0*.*0649*

### Association between collagen fragments in serum and clinical demographics and overall survival (OS) outcome

First, when evaluating the association between stage of disease and Karnofsky performance status (KPS) and collagen fragments, serum C1M and PRO-C3 were significantly higher in patients with Stage IV vs Stage II and III disease (Supplementary Table 2). Similarly, serum C1M, C3M, and PRO-C3 were significantly higher in patients with a KPS of 1 vs 0.

When evaluated by univariate analysis (Table 3), C3M, C4M and PRO-C3 predicted for significantly shorter OS when evaluated on a continuous scale (*p* = *0*.*004–0*.*037*), while C1M was only borderline significant (*p* = *0*.*056*). In contrast, the C3M/PRO-C3 ratio predicted for improved OS (*p* = *0*.*035*). When evaluating the four collagen fragments individually on a categorical scale by dichotomization at the 25^th^ percentile cut-point, patients above the 25^th^ percentile cut-point had shorter OS. Patients had an approximately 1.5-fold increased risk of dying if presenting with high levels of C1M (HR = 1.54, *p* = *0*.*0*25), C3M (HR = 1.44, *p* = *0*.*053*) and C4M (HR = 1.47, *p* = *0*.*039*), respectively. Patients with high levels of PRO-C3 had a 2-fold increased risk of dying (HR = 2.01, *p* = *0*.*001*). Again, high C3M/PRO-C3 predicted for a survival benefit with ratio-high patients having a 47% reduced risk of dying (HR = 0.53, *p* = *0*.*002*).

**Table 3:** Univariate Cox proportional hazards modelling for evaluating the association between serum collagen fragments (C1M, C3M, C4M and PRO-C3) and overall survival (OS) in patients with advanced unresectable PDAC.

Biomarker	HR	95% CI	*p-value*
***Continuous scale (n***, ***events/total)***			
C1M (16/149)	1.002	0.999–1.005	*0*.*056*
C3M (17/152)	1.019	1.004–1.036	*0*.*015*
C4M (17/152)	1.007	1.0004–1.013	*0*.*037*
PRO-C3 (16/149)	1.001	1.006–1.014	*0*.*004*
C3M/PRO-C3 (ratio) (16/149)	0.770	0.610–0.982	*0*.*035*
***Categorical scale*, ≤*25***^***th***^ ***vs*. >*25***^***th***^ ***percentile***			
C1M	1.54	1.06–2.25	*0*.*025*
C3M	1.44	0.96–2.1	*0*.*053*
C4M	1.47	1.02–2.15	*0*.*039*
PRO-C3	2.01	1.33–3.05	*0*.*001*
C3M/PRO-C3	0.53	0.35–0.80	*0*.*002*

**Figure 2:**
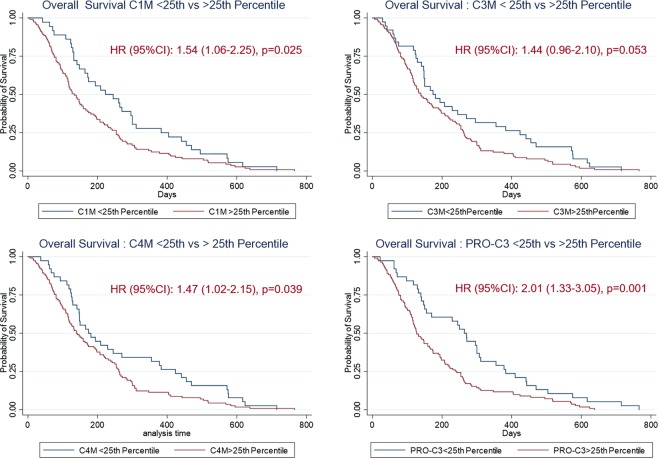
Probability of overall survival (OS) evaluated by Kaplan-Meier plots of patients with PDAC after dichotomizing the individual collagen biomarkers (C1M, C3M, C4M, PRO-C3) at the 25^th^ percentile cut-point. Hazard Ratio (HR) with 95% confidence interval (95%CI) for patients with biomarker levels >25^th^ percentile shown on each graph.

Next, an association between the collagen fragments and OS was evaluated by Kaplan-Meier plots using the 25^th^ percentile cut-point (Fig. 2). Overall, high levels of serum collagen fragments were associated with shorter OS. Median OS for patients with high vs low (>25% vs ≤25%) C1M was 133 days vs. 223 days; high vs low C3M was 139 days vs. 174 days; high vs low C4M was 139 days vs. 174 days, and high vs low PRO-C3 was 127 days vs. 264 days. When evaluating the C3M/PRO-C3 ratio, median OS for patients with a high C3M/PRO-C3 ratio was 172 days vs 109 days for patients with a low C3M/PRO-C3 ratio (not shown).

Lastly, multivariate analysis using the Cox proportional hazards model was used to evaluate the predictive value of the serum collagen fragments and clinical demographics with OS (Table 4). The serum collagen fragments were analyzed by dichotomization at the 25^th^ percentile cut-point, and clinical demographics included age (continuous), stage (categorical) and Karnofsky performance status (KPS) (categorical). In this multivariate analysis (Table 4) (adjusted for serum CA19-9), only serum PRO-C3 (HR 1.65, p = 0.018), and stage (HR 1.33, p = 0.03) remained significant. Age, KPS, and the remaining serum collagen fragments (C1M, C3M, and C4M) were not significant.

**Table 4:** Multivariate Cox proportional hazards modelling adjusted for serum CA19-9 for evaluating the predictive value of serum collagen fragments (C1M, C3M, C4M and PRO-C3) and clinical demographics with overall survival (OS) in patients with advanced unresectable PDAC.

Biomarker	HR	95% CI	*p-value*
Age (continuous)	1.003	0.986–1.021	*0*.*704*
Stage (II + III vs IV)	1.33	1.03–1.71	*0*.*030*
KPS (0 vs 1)	0.79	0.44–1.40	*0*.*418*
C1M, 25^th^ percentile	1.03	0.64–1.67	*0*.*893*
C3M, 25^th^ percentile	1.08	0.47–2.49	*0*.*856*
C4M, 25^th^ percentile	1.27	0.57–2.86	*0*.*558*
PRO-C3, 25^th^ percentile	1.65	1.09–2.48	*0*.*018*

## Discussion

PDAC is the most stroma-rich cancer type characterized by severe desmoplasia and ongoing ECM remodeling, both in the primary tumor and metastatic lesions^7^. Alterations in the tumor ECM not only associates with aggressive disease and impedes access/activity of anti-tumor therapy, but also generates circulating collagen fragments with pathologic fingerprints. In this study we measured such fingerprints in association with OS outcome.

The present results show that specific collagen peptide fingerprints that reflect collagen synthesis (PRO-C3) and collagen degradation (C1M, C3M, C4M) are highly elevated in patients with advanced unresectable PDAC compared to the healthy reference range, have a relatively broad dynamic range and associate with outcome when measured in serum prior to treatment with 5-FU-based therapy. These findings support what has been reported with the same markers in diagnostic (case-control) studies of lung cancer, breast cancer and ovarian cancer as well as prognostic studies of both metastatic breast cancer patients treated with letrozole or trastuzumab, and metastatic melanoma patients treated with ipilimumab^34–37^. Importantly, this study also confirms previous findings showing that MMP-mediated collagen turnover measured in serum by C1M, C3M and C4M is elevated in PDAC compared to healthy controls, as well as data showing prognostic value of PRO-C3 measured in PC patients^26,38^.

Of the four collagen markers tested, PRO-C3 and the C3M/PRO-C3 ratio performed best in predicting OS outcome in PDAC. PRO-C3 measures the pro-peptide that is released when type III collagen assembles in the tissue. Type III collagen is synthesized by activated fibroblasts and is a major component of the desmoplastic reaction and ECM accumulation in the tumor. C3M is a measure of type III collagen degradation, therefore the C3M/PRO-C3 ratio can be interpreted to reflect a reduced net fibrotic activity: high collagen degradation (C3M) with low collagen accumulation (PRO-C3). Clearly, this supports the notion that by measuring the surrogates of the desmoplastic reaction and altered turnover of the interstitial collagens (e.g. type III collagen), it is possible to identify patients with worsened prognosis^39^. In addition, PRO-C3 did not associate with CA19-9, indicating that PRO-C3 reflects a different underlying biology than the current gold standard PDAC serum biomarker (CA19-9), and hence may add additional value when evaluated to a greater extent in future clinical trials as they are produced by different cell types in the tumor microenvironment, i.e. cancer cells (CA19-9) and cancer associated fibroblasts (PRO-C3).

Previous findings have shown that collagen types I, III, and IV are concomitantly overexpressed together with hyaluronan (HA, a high-molecular-mass polysaccharide found in the ECM), predicting for poor prognosis and poor response to chemotherapy in PC^7,40^. This ultimately led to development of a recombinant pegylated hyaluronidase enzyme (PEGPH20, targeting HA), which would potentially increase drug uptake of chemotherapy^41^. A randomized phase II study (HALO-202) of PEGH20 combined with gemcitabine/nab-paclitaxel in stage 4 PDAC patients showed promising results of adding PEGPH20 to chemotherapy in a subgroup of patients with HA-positive tumor biopsies^42^. Interestingly, preliminary data based on retrospective analysis of HALO-202 also suggested that the C3M/PRO-C3 ratio was able to identify a subgroup of patients with better benefit of PEGPH20^43^. Together these findings suggest not only prognostic potential of C3M and PRO-C3, but also the ability to predict the likelihood of responding to a given combination of modalities. With the next wave of stroma/ECM-targeting therapy for PDAC on its way, inclusion of non-invasive biomarkers to identify specific subtypes of PDAC with ongoing desmoplasia is an important step forward^44–46^. However, to include the present biomarkers in clinical trial decision-making, the present study needs to be validated in larger, well characterized clinical cohorts, since the present result is limited by a relatively small sample size with limited available clinical information and is a retrospective biomarker analysis for predicting outcome.

In conclusion, this study shows that collagen remodeling biomarkers measured in pre-treatment serum are highly elevated and associated with OS in PDAC patients, supporting the link between the desmoplastic reaction, tumorigenesis, prognosis, and response to treatment. Of the four biomarkers evaluated, PRO-C3, a biomarker of type III collagen formation, appeared to provide the best prognostic value, independent of CA19-9. In summary, these results aid in the understanding of ECM turnover (formation and degradation of different collagens) as an important component in tumor progression and/or response to treatment. If validated in larger clinical cohorts, these collagen biomarkers may have potential as prognostic and/or predictive biomarkers in the PDAC setting.

## Supplementary information

Supplementary Information 

## Data Availability

The datasets used and/or analysed during the current study are available from the corresponding author on reasonable request.
