# Supplementary material for: Collagen fragments quantified in serum as measures of desmoplasia associate with survival outcome in patients with advanced pancreatic cancer
Source: Sci Rep. 2019 Dec 24;9:19761. doi: 10.1038/s41598-019-56268-3 (PMC6930304; doi:10.1038/s41598-019-56268-3)
Supplement: Supplementary file 1 — Supplementary Information [file 41598_2019_56268_MOESM1_ESM.docx]

Supplementary to manuscript: Collagen fragments quantified in serum as measures of desmoplasia associate with survival outcome in patients with advanced pancreatic cancer

Nicholas Willumsen^1^, Suhail M. Ali^2,3^, Kim Leitzel^2^, Joseph J. Drabick^2^, Nelson Yee^2^, Hyma V Polimera^2^, Vinod Nagabhairu^4^, Laura Krecko^2^, Ayesha Ali^2^, Ashok Maddukuri^2^, Prashanth Moku^2^, Aamnah Ali^2^, Joyson Poulose^2^, Harry Menon^2^, Neha Pancholy^2^, Luis Costa^5,6^, Morten A. Karsdal^1^, Allan Lipton^2^

^1^Biomarkers & Research, Nordic Bioscience, Herlev, Denmark, ^2^Department of Medicine, Penn State Health Milton S Hershey Medical Center, Hershey, PA, USA,^3^Hematology & Oncology Lebanon VA Medical Center, Lebanon, PA, USA, ^4^Pinnacle Health System, University of Pittsburgh Medical Center, Harrisburg, PA; USA,^5^Oncology division, Hospital de Santa Maria, Lisboa, Portugal,^6^Clinical Translational Oncology Research Unit, Institute of Molecular Medicine, Lisboa, Portugal

Supplementary Table 1

Clinical demographics and biomarker levels:

| **Variable** | **Mean** | **Min** | **Max** |
| --- | --- | --- | --- |
| Height (m) | 1.69 | 1.47 | 1.93 |
| Weight (Kg) | 65.47 | 32.73 | 106.36 |
| Age (yrs) | 64.52 | 36.56 | 86.78 |
| BSA | 22.80 | 13.20 | 35.26 |
| C1M, ng/ml | 45.13 | 15.05 | 362.85 |
| C3M, ng/ml | 9.92 | 6.00 | 58.69 |
| C4M, ng/ml | 23.87 | 3.07 | 122.98 |
| PRO-C3, ng/ml | 34.72 | 5.04 | 201.79 |

Supplementary Table 2

Stage and Karnofsky performance status (KPS) and biomarker levels:

|  | **Stage*** | | | | **KPS** | | |
| --- | --- | --- | --- | --- | --- | --- | --- |
| **variable** | **II** | **III** | **IV** | **p-value^** | **0** | **1** | **p-value** |
| C1M | 43.0 | 42.7 | 64.0 | 0.002 | 55.0 | 84.6 | 0.013 |
| C3M | 21.4 | 23.7 | 22.3 | 0.480 | 21.7 | 27.9 | 0.030 |
| C4M | 51.4 | 53.4 | 51.3 | 0.400 | 49.5 | 69.6 | 0.014 |
| PRO-C3 | 17.6 | 20.8 | 33.2 | 0.020 | 27.8 | 41.8 | 0.080 |

* mean ECM fragment serum level (ng/ml)

^ Stage II and III vs IV
